# Supplementary material for: The Role of Aquaporin Overexpression in the Modulation of Transcription of Heavy Metal Transporters under Cadmium Treatment in Poplar
Source: Plants (Basel). 2020 Dec 29;10(1):54. doi: 10.3390/plants10010054 (PMC7824648; doi:10.3390/plants10010054)
Supplement: Supplementary file 1 [file plants-10-00054-s001.pdf]

**Supplementary material**

# The role of aquaporin overexpression in the modulation of transcription of heavy metal transporters under cadmium treatment in poplar

**Andrea Neri[1], Silvia Traversari[1], Andrea Andreucci[2*], Alessandra Francini[1*] and Luca Sebastiani[1]**

[1]BioLabs, Institute of Life Sciences, Scuola Superiore Sant'Anna, Piazza Martiri della Libertà 33, Pisa, Italy.
[2]Department of Biology, University of Pisa, via Luca Ghini 13, Pisa, Italy.
* Correspondence: author e-mails, andrea.andreucci@unipi.it; a.francini@santannapisa.it

**Table S1** List of primers used for the gene transcription analyses by RT-PCRs.

| Gene | Primer sequence 5'-3' | Amplicon size (bp) |
|---|---|---|
| *PaABCC9* | Forward: GCAGGACTTTCAACAGCACA<br>Reverse: TTTAATATCCTTCCGGTTGGGG | 100 |
| *PaHMA2* | Forward: CTTTGCTCTTATAGTGCCGACAA<br>Reverse: ATGGCCTCTGCAACCCCT | 150 |
| *PaABCC13* | Forward: GCTCCCATGCTATTTTTCCACACT<br>Reverse: ACAATGCCTATCAGCACAAAAGTTG | 165 |
| *PaNRAMP3.1* | Forward: GTTGGAGTTGTGGGTTGCAT<br>Reverse: CGAACGATATTACAAGGGCAGTG | 150 |
| *PaNRAMP3.2* | Forward: TTTGGACCCTGGCAATTTGG<br>Reverse: CAGCCATAATCCACAAAATCATTCG | 208 |
| *PaNRAMP1.3* | Forward: GGAGCGCAATAAAGTATGAGTTAC<br>Reverse: CAATTTCAGCAAGGACCCACAAG | 180 |
| *PaNRAMP2* | Forward: CCGTTGATTATGTCCCACCATTC<br>Reverse: GCCATAACAACGAGTACCCA | 145 |
| *18S* | Forward: AATTGTTGGTCTTCAACGAGGAA<br>Reverse: AAAGGGCAGGGACGTAGTCAA | 145 |

**Table S2** Two-way ANOVA results of Cd, Zn and Mn concentrations: ns (not significant), * (0.01 < *P* < 0.05), ** (0.001 < *P* < 0.01), and *** (*P* < 0.001).

| | | ***Cd*** | | | ***Zn*** | | | ***Mn*** | | |
|---|---|---|---|---|---|---|---|---|---|---|
| Organ | Days | Cd | Line | Cd × Line | Cd | Line | Cd × Line | Cd | Line | Cd × Line |
| *Root* | | *** | ns | ns | ns | ns | ns | ns | ns | * |
| *Stem* | 1 | *** | ns | ns | ns | ns | ns | ns | ns | ns |
| *Leaves* | | ns | ns | ns | ns | ns | ns | ns | ns | ns |
| *Root* | | *** | ns | ns | ns | ns | ns | *** | ** | ** |
| *Stem* | | *** | ns | ns | ns | ns | ns | * | ns | ns |
| *Basal leaves* | 7 | ns | ns | ns | ns | ns | ns | ns | ns | * |
| *Medial leaves* | | *** | *** | *** | ns | ns | ns | ns | ns | ns |
| *Apical leaves* | | *** | ns | ns | ** | ns | ns | ** | ** | ** |
| *Root* | | *** | ns | ns | ns | ns | ns | *** | ns | ns |
| *Stem* | | *** | ns | ns | * | ns | ns | *** | ns | ns |
| *Basal leaves* | 60 | *** | ns | ns | ns | ns | ns | ** | ns | *** |
| *Medial leaves* | | *** | ** | ** | ns | ns | ns | * | ns | ns |
| *Apical leaves* | | *** | ns | ns | ns | ns | ns | ns | ns | ns |

**Table S3** Two-way ANOVA results of studied genes: ns (not significant), * (0.01 < *P* < 0.05), ** (0.001 < *P* < 0.01), and *** (*P* < 0.001).

| ***Parameter*** | ***Organ*** | ***Days of treatment*** | ***Cd*** | ***Line*** | ***Cd × Line*** |
|---|---|---|---|---|---|
| ***HMA2*** | *Root* | 1 | ** | *** | ** |
| | *Stem* | | ** | *** | ** |
| | *Leaves* | | ** | *** | ** |
| ***NRAMP3.1*** | *Root* | 1 | *** | ns | * |
| | *Stem* | | *** | ns | * |
| | *Leaves* | | *** | ns | * |
| ***NRAMP3.2*** | *Root* | 1 | ns | ns | * |
| | *Stem* | | ** | * | * |
| | *Leaves* | | *** | *** | *** |
| ***NRAMP1.3*** | *Root* | 1 | ns | ns | ns |
| | *Stem* | | ns | ns | ns |
| | *Leaves* | | ns | ns | ns |
| ***NRAMP2*** | *Root* | 1 | ns | ns | ns |
| | *Stem* | | *** | ** | ** |
| | *Leaves* | | ns | ns | ** |
| ***ABCC9*** | *Root* | 1 | * | ** | * |
| | *Stem* | | ns | ns | ns |

| | | | | | |
|---|---|---|---|---|---|
| | *Leaves* | | * | *** | * |
| ***ABCC13*** | *Root* | 1 | * | ns | ns |
| | *Stem* | | * | * | * |
| | *Leaves* | | ns | ns | ** |
| ***HMA2*** | *Root* | 7 | ns | * | * |
| | *Stem* | | ns | * | * |
| | *Basal leaves* | | ns | * | * |
| | *Medial leaves* | | ns | * | * |
| | *Apical leaves* | | ns | * | * |
| ***NRAMP3.1*** | *Root* | 7 | ns | * | ns |
| | *Stem* | | ns | * | * |
| | *Basal leaves* | | ns | ns | ns |
| | *Medial leaves* | | ns | * | ns |
| | *Apical leaves* | | ns | ns | ns |
| ***NRAMP3.2*** | *Root* | 7 | ns | ns | ns |
| | *Stem* | | ** | * | ** |
| | *Basal leaves* | | ns | * | ns |
| | *Medial leaves* | | ns | * | ns |
| | *Apical leaves* | | ns | ns | ns |
| ***NRAMP1.3*** | *Root* | 7 | ns | ns | ns |
| | *Stem* | | ns | ns | * |
| | *Basal leaves* | | ns | ns | ns |
| | *Medial leaves* | | ns | ns | ns |
| | *Apical leaves* | | ns | ns | * |
| ***NRAMP2*** | *Root* | 7 | ** | ns | * |
| | *Stem* | | ns | ns | ns |
| | *Basal leaves* | | ns | ns | ns |
| | *Medial leaves* | | ns | ns | ns |
| | *Apical leaves* | | ns | * | ** |
| ***ABCC9*** | *Root* | 7 | ns | * | ns |
| | *Stem* | | * | ns | ** |
| | *Basal leaves* | | ns | ns | ns |
| | *Medial leaves* | | ns | ns | ns |
| | *Apical leaves* | | ns | ns | ns |
| ***ABCC13*** | *Root* | 7 | ns | ns | ns |
| | *Stem* | | * | ns | ns |
| | *Basal leaves* | | ** | ns | ** |
| | *Medial leaves* | | ns | ns | ns |
| | *Apical leaves* | | ns | ns | ns |
| ***HMA2*** | *Root* | 60 | ns | ns | ns |

| | | | | | |
|---|---|---|---|---|---|
| | *Stem* | | ns | ns | ns |
| | *Basal leaves* | | ns | ns | ns |
| | *Medial leaves* | | * | ns | * |
| | *Apical leaves* | | ns | ns | ns |
| ***NRAMP3.1*** | *Root* | 60 | ns | ** | ns |
| | *Stem* | | * | * | ns |
| | *Basal leaves* | | * | ns | * |
| | *Medial leaves* | | ns | ns | ns |
| | *Apical leaves* | | *** | * | ns |
| ***NRAMP3.2*** | *Root* | 60 | ns | ns | ns |
| | *Stem* | | *** | ** | ns |
| | *Basal leaves* | | ns | ns | ns |
| | *Medial leaves* | | ns | ns | ns |
| | *Apical leaves* | | * | * | ns |
| ***NRAMP1.3*** | *Root* | 60 | * | ns | ns |
| | *Stem* | | *** | *** | ** |
| | *Basal leaves* | | ns | * | ns |
| | *Medial leaves* | | ns | ns | ns |
| | *Apical leaves* | | ns | ns | ns |
| ***NRAMP2*** | *Root* | 60 | * | ns | ns |
| | *Stem* | | ns | * | ns |
| | *Basal leaves* | | ns | ns | ns |
| | *Medial leaves* | | ns | ns | ns |
| | *Apical leaves* | | * | ns | ns |
| ***ABCC9*** | *Root* | 60 | ns | ns | ns |
| | *Stem* | | *** | ** | ** |
| | *Basal leaves* | | ns | ns | ns |
| | *Medial leaves* | | ns | ns | ns |
| | *Apical leaves* | | ns | ns | ns |
| ***ABCC13*** | *Root* | 60 | ns | ns | ns |
| | *Stem* | | * | * | * |
| | *Basal leaves* | | ns | ns | ns |
| | *Medial leaves* | | ns | ns | ns |
| | *Apical leaves* | | ** | ns | ** |

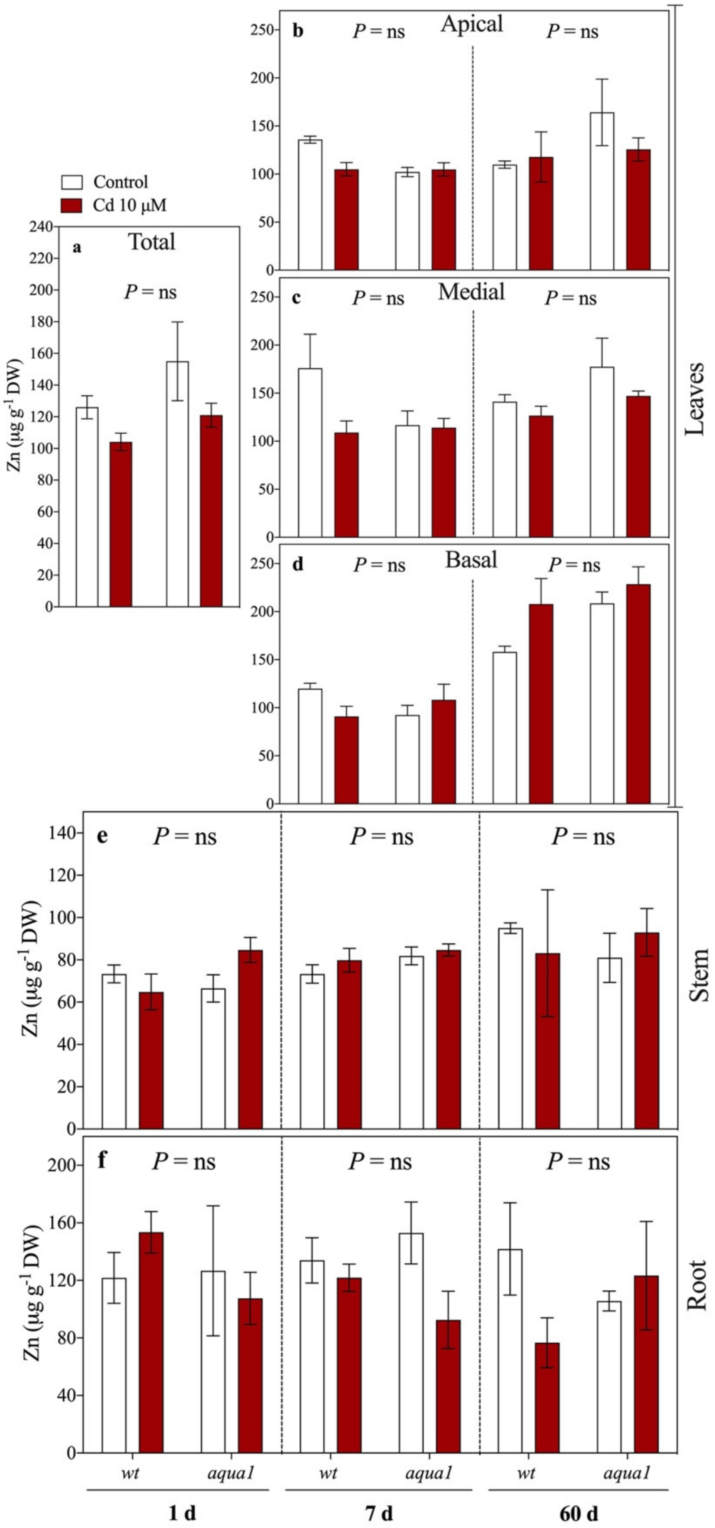


**Fig. S1** Zn concentrations (μg $g^{-1}$ DW) in leaves (a-d), stem (e), and root (f). In the first sampling time (1 d) the leaves were not divided in three groups (a) while at 7 and 60 d leaves were divided in three groups: apical (b), medial (c), and basal (d). Values represent the mean of three biological replicates ± SD. Data were analysed with two-way ANOVA; *P* values correspond to the interaction between Cd and Line are reported in the figure (ns = not significant). When the interaction was significant, different letters indicate significant differences among treatments at each sampling time and organ. Cd and Line *P* values are reported in Table S2.

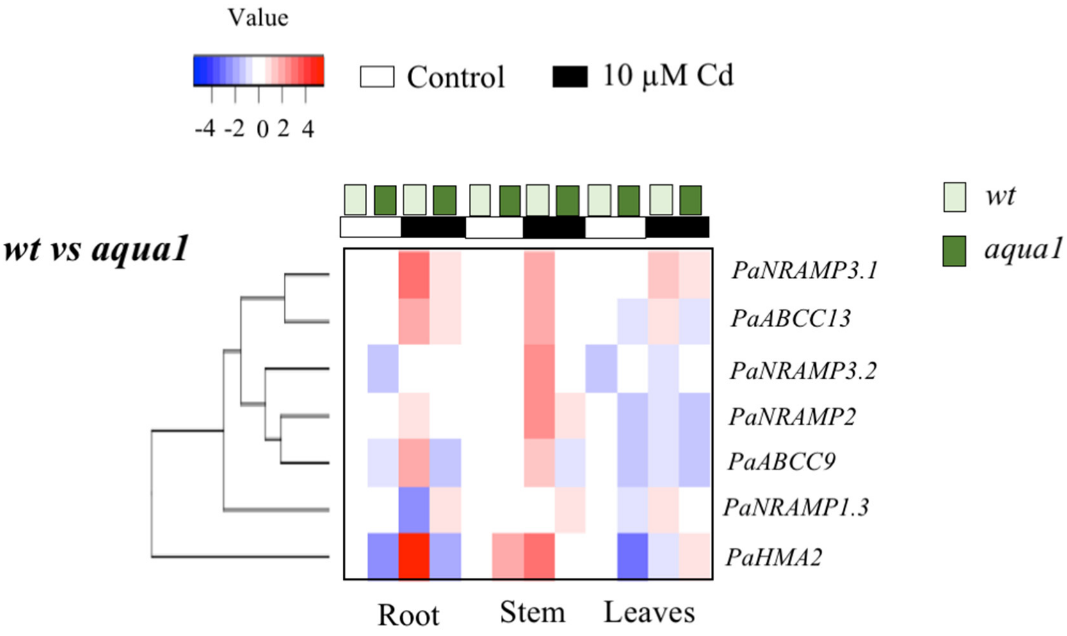


**Figure S2.** Heat map of the comparison between *wt* and *aqua1* gene transcription levels in control and treated conditions after 1 d of Cd treatment. Data displayed in the heat map are expressed in $\ln 2^{-\Delta\Delta Ct.}$
